# Multicenter prospective evaluation of a new articulating 5-mm endoscopic linear stapler

**DOI:** 10.1007/s00464-015-4406-4

**Published:** 2015-07-25

**Authors:** Andreas Kuthe, Alexander Haemmerle, Kaja Ludwig, Stephan Falck, Wolfgang Hiller, Frederick Mainik, Stephan Freys, Lev Dubovoy, Joachim Jaehne, Karl Oldhafer

**Affiliations:** DRK‐Krankenhaus Clementinenhaus, Allgemein-, Viszeral- und Unfallchirurgie, Lützerodestr. 1, 30161 Hannover, Germany; Sana Klinikum Hameln‐Pyrmont, Klinik für Allgemein- und Visceralchirurgie, Saint-Maur-Platz 1, 31785 Hameln, Germany; Klinikum Suedstadt Rostock, Klinik für Allgemein- und Visceralchirurgie, Sudring 81, 18059 Rostock, Germany; Asklepios Klinik Barmbek, Allgemein-, Viszeralchirurgie, Rübenkamp 220, 22291 Hamburg, Germany; DIAKO Ev. Diakonie‐Krankenhaus, Klinik für Allgemein-und Visceralchirurgie, Gröpelinger Heerstraße 406 ‐ 408, 28239 Bremen, Germany; Klinikum Lippe, Allgemein- und Viszeralchirurgie Detmold, Röntgenstraße 18, 32756 Detmold, Germany; Henriettenstiftung Hannover, Klinik für Allgemein- und Visceralchirurgie, Marienstraße 72-90, 30171 Hannover, Germany

**Keywords:** Laparoscopy, Staples, Surgical stapler, Anastomosis, Laparoscopic surgery

## Abstract

**Background:**

The objective of this study was to evaluate the safety and efficacy of a novel 5-mm laparoscopic linear stapler in clinical gastrointestinal surgical applications.

**Methods:**

A prospective, single-arm study with an open enrollment of subjects requiring stapling of the gastrointestinal (GI) tract was performed. The study endpoints were the number of complications and technical failures associated with the use of a novel stapler when compared to similar events with conventional staplers as described in the medical literature.

**Results:**

Seven centers enrolled 160 subjects, 150 of which were followed up to at least 30 days postoperatively. Intraoperative success: In 423 deployments, there were two staple line leaks and five staple line bleeds, all of which were intraoperatively resolved. In addition, incomplete staple lines were noted as a result of user error (*n* = 15) or device-related issues (*n* = 22), all of which were immediately resolved and none of which resulted in a complication or a change of the surgical procedure. Late outcomes: A total of 13 surgical complications in 160 patients were related to a GI transection or anastomosis, 12 of which related to a hand-sewn anastomosis or use of other commercially available staplers. One event (1/153, 0.065 %) on POD 1, involving bleeding of the staple line, was felt to be related to the use of the new stapler.

**Conclusion:**

The study confirmed that the new device was user-friendly (9 % incidence of problems firing the device), reliable (3 % device failures) and safe (<1 % complication rate related to the stapler). Based on these results, it would seem that this new 5-mm stapler is a safe and effective alternative to standard 12-mm staplers.

One of the great advancements in patient care in the last 30 years has been the move away from massive open surgical incisions toward more minimal access, image guided and organ sparing surgeries. The introduction of laparoscopic and thoracoscopic surgery in particular has resulted in billions of healthcare dollars saved due to fewer wound complications, shortened hospital stays, lessened late bowel obstructions and faster return to normal productivity. The success of laparoscopic surgery and evolution of imaging technology have led to continuing efforts to further reduce access trauma by reducing the number and size of the access ports. Reduction in access port size has been shown to reduce postoperative complications such as wound infection, abdominal herniation, pain and disfiguring scars, and today, the 5-mm access port is the most common size for the majority of abdominal and thoracic surgeries [1–6].

The introduction of laparoscopic versions of surgical staplers in the early 1990s is deemed to be one of the key technologic developments that most enabled the widespread application of minimally invasive surgery. As stapling had largely supplanted suturing for most GI tract resections, anastomosis and vascular and pulmonary divisions before the advent of thoracoscopy and laparoscopy, and as video-assisted suturing is considered technically difficult to master, staplers that fit through trocar ports were essential to advance these minimally invasive procedures beyond cholecystectomy. Conventional staplers were modified in the early 1990s to enable them to fit through available 12 mm and larger trocars. Because these staplers for standard size staples (white, blue and green) were adaptations of existing open staplers, it has proven to be impossible to reduce their working diameter below 12 mm in order to fit through more modern ports of 3–10 mm.

## Materials and methods

The study protocol, information/consent form and any materials used to recruit subjects were approved by independent ethics committees at each hospital [7].

All subjects signed an informed consent that contained information regarding the purpose, procedures, requirements and restrictions of the study along with any known risks and potential benefits, any available compensation and the established provisions for confidentiality. Subjects also were informed that they could withdraw from the study at any time for any reason and could receive an alternate form of therapy.

### Study design

The study was a prospective, single-arm, multicenter study with an open enrollment of any subject requiring gastrointestinal procedures. The objective was to document the safety and efficacy of the new stapler and demonstrate non‐inferiority of the MicroCutter to conventional staplers based on historical failure rates in the literature. Concomitant use of conventional staplers (Covidien Inc., Mansfield, MA; Ethicon Endo-Surgery Inc., Cincinnati, OH) would allow an additional comparison of stapler-related adverse events within study subjects.

All subjects who were candidates for surgery where the use of a linear stapler was anticipated for visceral division or anastomosis were considered eligible for enrollment in this study. There were no preoperative inclusion or exclusion criteria.

### Data collection

The investigators maintained detailed records on all study subjects; study-specific data were recorded in the subject’s charts and entered onto case report forms. Preoperative assessment included the subject’s medical history, presenting symptoms, physical status, American Society of Anesthesiologists (ASA) classification) [8] and any preoperative medication that might affect wound healing or bleeding.

Intraoperative data collection included the surgical procedure, the type and size of access, the need for conversion in laparoscopic procedures, any use of conventional stapling technology, any use of hand suture techniques and all information related to MicroCutter deployments such as the frequency and localization of the deployment, and the success of each deployment as described in more detail below. Pre-discharge data included the need for and length of stay in intensive care, the need for antibiotic therapy or blood transfusions, any complications, any symptoms related to the surgical procedure and the overall length of stay.

Subjects were asked to return for a follow-up examination within 30 days after surgery. If the subject could not report to the follow-up in person, a follow-up interview was conducted by phone. The 30-day follow-up evaluation included a determination of whether the subject had been re-hospitalized between discharge and the 30-day follow-up. Any hospitalizations were recorded separately with the date, duration of hospital stay and reason for hospitalization. All other complications related or not related to hospitalizations were also documented.

### Technology

**Fig. 1:**
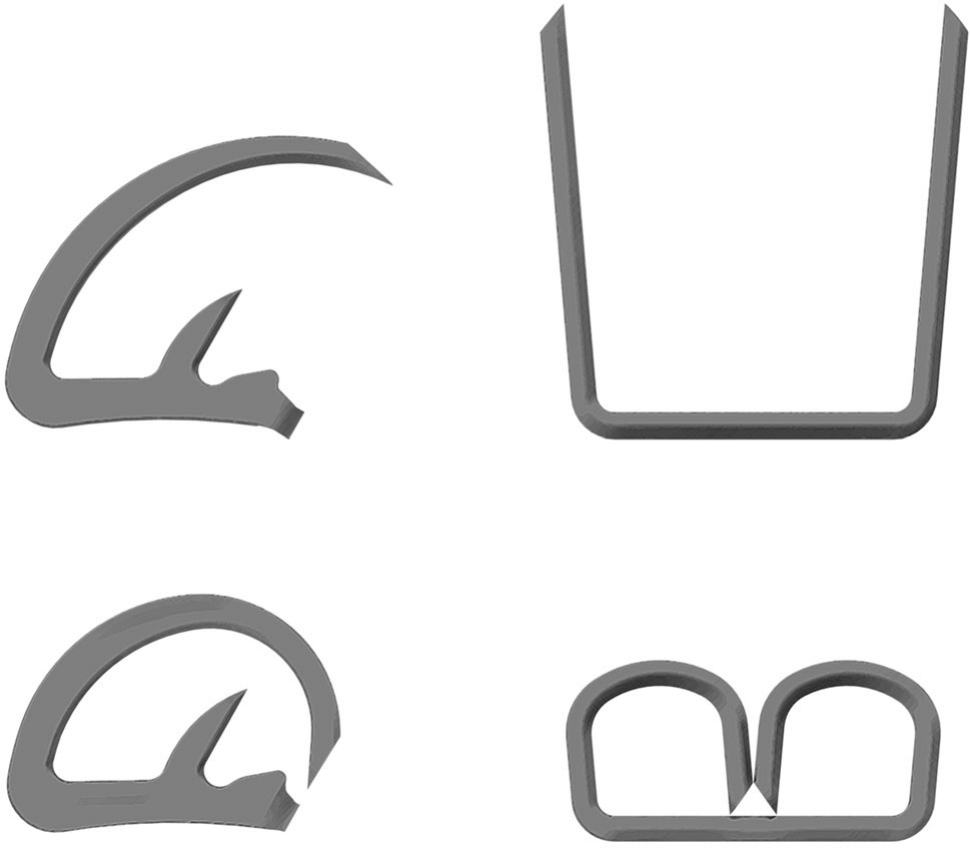
Comparison of “D-Shaped” (*left*) and “B-Shaped” (*right*) and staple forms. Pre-deployment forms are shown on the top and post-deployment forms are shown on the bottom

**Fig. 2:**
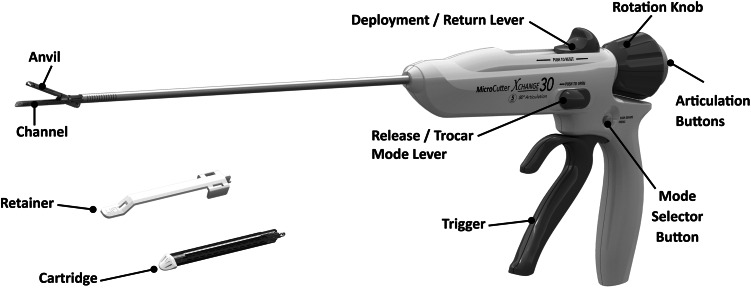
Technical description of MicroCutter XCHANGE 30

**Fig. 3:**
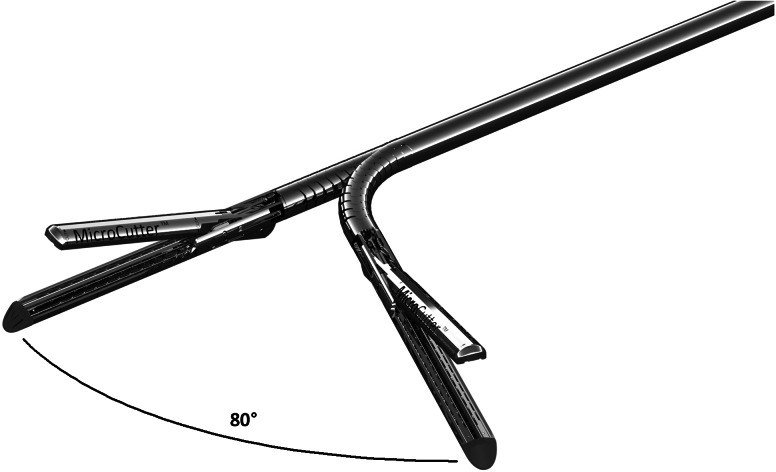
MicroCutter XCHANGE 30 End-effector in 80° articulation

In the current trial, the new stapler used blue cartridges. The stapler places 50 staples in 4 staggered rows with a linear cut in the center of the 4 rows. The small diameter of the stapler is made possible by using a new staple form, the “D” staple vs the traditional “B” staple [9] (Fig. 1). The stapler is a single-patient-use device (Fig. 2). The staple in the blue cartridge used in this study has a tine length of 3.43 mm and a crown or back span length of 1.88 mm. The overall closed-form height (outer diameter) is 1.4 mm, and the internal height at its apex is 0.875 mm. The stapler also allows articulation of the end‐effector to a maximum of 80° in either direction without touching the abdominal or thoracic wall for leverage (Fig. 3).

The D-shaped staples and MicroCutter stapler were CE (Conformité Européene) marked and later FDA cleared for human use.

### Surgical technique

Surgical approaches varied according to the procedure performed and by institutional preference. Indications for surgery, patient preparation, operative approaches, either open or laparoscopic/thoracoscopic and postoperative care were not altered from standard practice at the participating institutions. Surgeons were allowed free use of standard 12-mm staplers or the new 5-mm stapler at their discretion. Patients were blinded to the use or not of the new stapler.

### Study endpoints

The primary study outcome was the safety and efficacy of the new stapler as determined by the incidence of severe adverse events up to 30 days postoperatively. The incidence of complications (composite of infection, leakage, bleeding and strictures) was compared to a composite conventional stapler-related adverse event rate as derived from a comprehensive analysis of the medical literature.

The secondary study endpoint was acute procedural success with the MicroCutter for each deployment during surgery. Acute procedural success was defined by:

- *Ability to access the target site*—ability to insert the device through a trocar 5 mm or larger, articulate or rotate the shaft, and position the tissue into the jaws
- *Completeness of the staple line*—ability to fully deploy all staples to complete a 30-mm staple line, where all staples have formed completely, no staples are missing from the target tissue and the device is able to be reset, unclamped and removed from the target tissue.
- *Completeness of the stapler cut*—ability of the device to cut through the tissue clamped in the jaws during staple deployment up to the point where staples have been deployed.
- *Absence of immediate staple line leakage*—the unintended passage of bowel fluids or air across the staple line.
- *Absence of immediate staple line bleeding*—pulsatile bleeding or bleeding that requires an intervention such as placement of a stitch or clip at the bleeding site.
- *Need for surgical intervention*—need for a stitch, second staple line or clip as a result of staple line bleeding, staple leakage/dehiscence, staple line stricture or tissue transection without staple line placement

### Historical controls based on a review of the medical literature

Surgical stapling is typically associated with four serious adverse events: stapling line leakage or dehiscence, staple line bleeding, staple line infection and staple line strictures. Sometimes these occurrences are the fault of the stapler, sometimes a factor of patient biology and sometimes multifactorial. As it is impossible in the literature to determine the reasons for failure, we chose to also record all problems with the MicroCutter staple lines as well in order to ensure a equitable comparison. A search was performed on the Medline database to determine the incidence of each of these adverse events in surgical subjects undergoing gastrointestinal procedures. In total, this analysis identified 58 recent peer‐reviewed medical publications citing incidences of any of the aforementioned adverse events within the perioperative and early postoperative period. These papers evaluated results from approximately 38,000 subjects.

A composite adverse event rate based on this review was calculated by adding the individual incidences. Based on this analysis, the composite adverse event rate for subjects undergoing a surgical procedure involving the use of a surgical stapler was 17.3 % (Table 1) [10–67].

**Table 1:** Composite adverse event ratio weighted average analysis from the medical literature

Adverse event	Studies	Patients	No. of SAE	Rate (%)	SD (%)	Min (%)	Max (%)
Infection	15	17,680	676	3.8	±5.6	0.7	19.7
Leakage	44	20,645	540	2.6	±2.4	0.0	12.7
Bleeding	17	5982	136	2.3	±1.4	0.8	5.9
Stricture	7	2262	195	8.6	±6.0	4.9	19.0

### Statistical methods

Based on a composite severe adverse event rate of 17.3 % and a non‐inferiority margin of 5 %, the MicroCutter would be considered to be non‐inferior if the upper 95 % confidence interval for the composite adverse event rate was less than 22.3 %. Based on the sample size calculations, a sample size of 160 subjects presenting at the 30-day visit would result in fulfilling the non‐inferiority requirement if the observed composite adverse event rate was less than or equal to 17.3 %.

## Results

One hundred and sixty (160) subjects were enrolled between July 2012 and May 2013 at 7 sites in Germany. Seventy procedures were performed via laparotomy (43.8 %), 75 (46.9 %) laparoscopically and 15 (9.4 %) as laparoscopic-assisted procedures. None of the laparoscopic or laparoscopic-assisted procedures were converted to open. The subject demographics are presented in Table 2.

**Table 2:** Subject demographics

Variable	Total (*n* = 160)
Age (years), mean ± SD	55.0 ± 18.6
BMI (kg/m^2^), mean ± SD	28.6 ± 10.9
Male, *n* (%)	73 (45.7)
History of smoking, *n* (%)	50 (32.5)
Diabetes mellitus, *n* (%)	27 (17.1)
Alcohol abuse, *n* (%)	8 (5.2)
Hyperlipidemia, *n* (%)	28 (27)
Hypertension, *n* (%)	71 (44.7)
Chronic lung disease, *n* (%)	25 (15.7)
Peripheral vascular disease, *n* (%)	8 (5)
Cerebrovascular accident, *n* (%)	10 (6.4)
History of coronary artery disease, *n* (%)	5 (5.7)
Hepatic failure, *n* (%)	4 (2.5)
Immunocompromised condition, *n* (%)	8 (5.4)
Bleeding disorder, *n* (%)	1 (0.7)
Preoperative symptoms	
Nausea, *n* (%)	31 (19.4)
Obstipation, *n* (%)	7 (4.4)
Diarrhea, *n* (%)	18 (11.3)
Pain, *n* (%)	63 (39.4)
ASA physical status	
Class 1	13 (8.2)
Class 2	65 (40.7)
Class 3	78 (48.8)
Class 4	4 (2.5)

### Surgical procedures

**Fig. 4:**
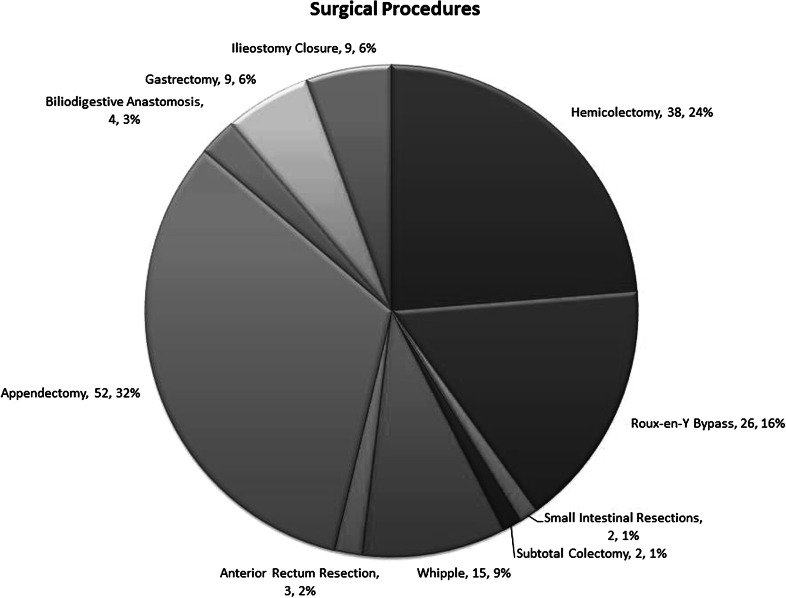
Surgical procedures

In this study, the MicroCutter was used in gastrointestinal procedures typically performed in general surgery. Figure 4 depicts the absolute numbers of procedures in each category as well as the relative percentages. The most commonly performed procedure was appendectomy (*n* = 52, 33 %), followed by hemicolectomy (*n* = 38, 24 %) and gastric bypass procedures (*n* = 26, 16 %).

The MicroCutter was deployed 423 times by 25 different surgeons. It was used to transect small intestine (*n* = 213, 50.4 %), colon 81 times (19.2 %), the appendix 57 times (13.5 %) and the duodenum 19 times (4.5 %). The MicroCutter was also used for anastomoses in 39 deployments (9.2 %), 25 times between the small intestine, 13 times between the small intestine and colon, and once to anastomose the small intestine to the stomach in a gastric bypass procedure. Less common uses of the MicroCutter were closures of enterotomy sites, transections of the common bile duct and transections of the mesocolon or the mesoappendix or to perform an oophorectomy. Deployments crossed previously placed staple lines in 160 of 423 (40 %) of total deployments.

Tissue outside the capable thickness range for the MicroCutter was transected using other commercially available staplers. Other stapling products were used in 42 % of the procedures and varied between sites as a function of surgeon preference, type of access (laparoscopic versus laparotomy) and types of procedures performed.

### Postoperative course and 30-day follow-up

Seventy-one subjects required intensive care stays with an average length of stay of 61 (19–93) hours. The need for intensive care unit care was a function of the complexity of the surgical procedures and was not related to the use of a stapler. One of the 160 subjects enrolled died prior to discharge (leakage of hand-sewn intestinal anastomosis). All 159 subjects discharged were questioned as to the presence of symptoms such as nausea, constipation, diarrhea or pain. The surgical wounds were examined at the time of discharge. Sixteen subjects had a wound issue after surgery. Approximately 8 % of the subjects complained of pain after the surgical procedure, 3 % of nausea, and approximately 1 % of constipation and 2 % of diarrhea. Antibiotic therapy was recorded if it was given outside the usual routine or prophylactic care. During the postoperative period, 22 subjects (13.8 %) received antibiotic therapy, and the majority of these therapies were indicated for wound infections. Twenty-one (13.2 %) subjects received a blood transfusion postoperatively. The average number of days between surgery and discharge was 9 days (0–43 days). Fourteen subjects did not undergo a formal physical examination (8.8 %) prior to discharge.

**Fig. 5:**
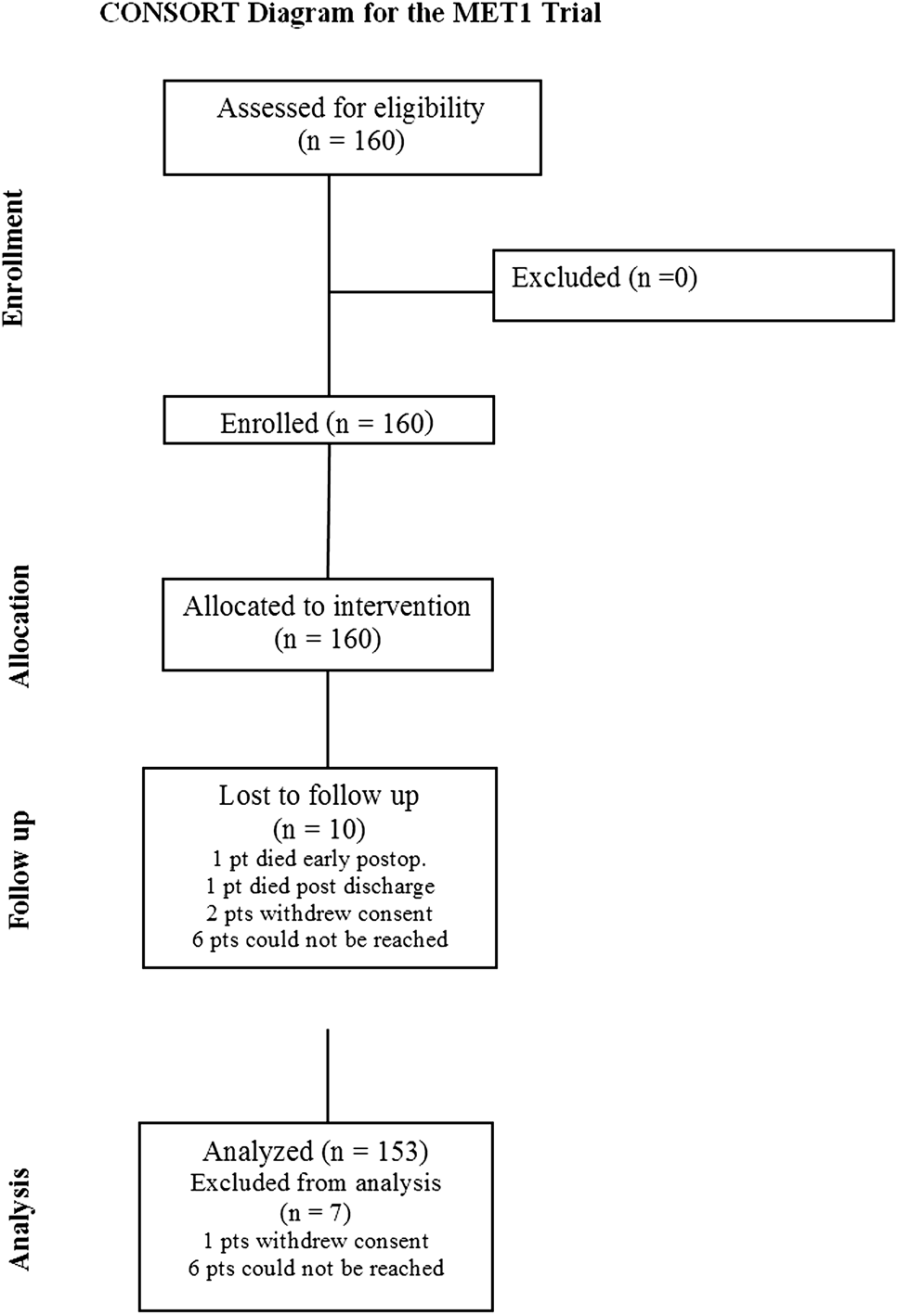
Consort Diagram

Of the 159 discharged patients, 150 (93.8 %) completed follow-up between 30 and 60 days postoperatively (Fig. 5). Forty percent of those subjects were seen in the clinic, and the remainder of subjects assessed by phone interview. Of the 10 subjects not available for follow-up, one had died prior to discharge (see above) and another prior to the 30-day follow-up. Of the remaining 8 subjects, 6 could not be reached despite numerous attempts and two subjects refused to be followed. Twenty-six re-hospitalizations were recorded in 24 subjects. Thirteen of these hospitalizations were associated with significant complications related to the surgery.

### Primary outcomes

A total of 36 (22 %) postoperative adverse events were reported. Twenty-three complications were unrelated to the use of any type of stapling device or hand-sewn anastomosis. These included general wound infections, general infections, ileus, neurological or other solid organ complications. Thirteen events were found to be related to the use of any type of stapler or hand-sewn anastomosis.

Six complications were related to hand-sewn anastomosis. Three of these six hand-sewn-related events were caused by anastomotic bleeding. One event was related to an infectious complication, and two were due to other complications related to the hand-sewn anastomosis.

Six complications were related to the use of other commercially available staplers that were used during the procedures. Two of these were leaks at the staple line. Another was related to a staple line-induced stricture at a gastrojejunostomy. Two were related to a staple line-related infectious complication at an anastomosis, and the last was a staple line complication, not otherwise defined.

There was one complication related to the use of the new stapler. This was a postoperative bleed from a small intestinal anastomosis made with the MicroCutter. The patient had undergone a laparoscopic right-sided hemicolectomy where the MicroCutter was used to transect the transverse colon (two deployments), ileum (two deployments) and to construct the ileocolic anastomosis (two deployments). All deployments were uneventful. At the time of surgery, the anastomosis was reported to be hemostatic and well perfused. The common enterotomy was closed using suture. In the early postoperative course, the subject presented with a drop in hemoglobin and hematochezia. The patient was brought back to the operating room within 24 h of the initial surgery, and a significant amount of blood in the intestine in proximity to the anastomosis was found. An arterial bleeder was seen at the distal end of the anastomotic staple line. The anastomosis was resected and a new anastomosis created. The subject recovered well.

One hundred and fifty subjects were followed at least 30 days postoperatively. Three subjects not followed for the full-time period had a complication and were therefore included in the denominator for the primary endpoint analysis. As the MicroCutter was presumed to be responsible for one complication, the incidence in relation to the number of followed patients at 30 days (*n* = 153) was 0.65 %.

The total composite staple-related severe adverse event rate from the medical literature in meta-analysis was 17.3 %. With one MicroCutter-related severe adverse event in 153 subjects followed at 30 days, the MicroCutter-related severe adverse event rate was 0.65 % (1/153^1^) with an exact upper 95 % confidence limit of 3.59 %. A non-inferiority analysis demonstrates non-inferiority of the MicroCutter when compared to stapler-related severe adverse event rates from the medical literature.

### Secondary outcomes

Intraoperative problems with the stapler were recorded and subsequently analyzed according to cause (Table 3).

**Table 3:** Stapler and hand-sewn anastomosis-related primary endpoint events

Stapler and hand-sewn-related severe adverse events categories	Hand-sewn related	Other stapler related	MicroCutter related	Total *N*
Leaks	0	2	0	2
Bleeding	3	0	1	4
Infections	1	2	0	3
Strictures	0	1	0	1
Other complications	2	1	0	3
Total	6	6	1	13

Based on the intraoperative assessments by the surgeons, 386 of 423 deployments (91.3 %) resulted in a perfect staple line. In the context of this study, this was defined as: all staples in the 30-mm staple line being fully deployed, with normal staple formation, no staples missing from the target tissue and the stapler able to be reset, unclamped and removed from the target tissue. Any “imperfect” deployments were evaluated, and each incident was classified as either device related (22 incidents) or user error (15 incidents).

Staple line leakage independent of an incomplete staple line was observed in two instances and intraoperatively resolved with either placement of a stitch (*n* = 1) or placement of a second 5-mm staple line (*n* = 1). Staple line bleeding was observed in five instances and intraoperatively resolved with placement of a stitch in four instances and by the use of electrocautery in one case (Table 4).

**Table 4:** Acute procedural success (secondary outcome)

Acute procedural success criteria	Secondary endpoint *N* (%) (based on investigation and analysis)	
	Met	Not met
Ability to access target site	423 (100)	0 (0)
Adequacy of the staple line	386 (91.3)	37 (8.7)
Completeness of the stapler cut	423 (100)	0 (0)
Presence or absence of immediate staple line leakage	421 (99.5)	2 (0.5)
Presence or absence of immediate staple line bleeding	419 (99.0)	4 (1.0)
Need for surgical intervention	422 (99.8)	1 (0.2)
Total		44 (10.4)

## Discussion

Surgical staplers have largely replaced traditional suture techniques throughout the Western world. Advanced laparoscopy in particular is dependent on the availability of staplers due to the perceived difficulties of laparoscopic suturing. The critical nature of the targets of surgical stapler usage, such as division of vascular structures, creation of anastomosis and sealing of bowel, makes the performance of these devices highly important to surgeons and to patient safety. The introduction of a new stapler must therefore be accompanied by proof that it is both effective (reliable and user-friendly) and safe. We report on a multicenter clinical outcomes study on a new 5-mm laparoscopic linear cutting stapler that assessed the acute and 30-day safety and efficacy of the MicroCutter 5-mm stapler. Results were compared to the safety and efficacy of standard laparoscopic staplers based on a meta-analysis of laparoscopic stapler studies (cumulative complication rate = 17 %). It was documented that, with a major complication rate of 0.65 %, the new stapler is as safe and effective as the currently available 12-mm staplers on the market.

The study population was representative of patients presenting to a tertiary GI surgery unit, and the procedures performed also represented the full range of typically performed general surgical procedures, from low-risk procedures (appendectomies) to high-risk procedures such as gastrectomies, Whipple operations or biliodigestive anastomoses. The distribution of low-risk (40 %), medium-risk (43 %) and high-risk procedures (19 %) performed in this trial represents a typical distribution encountered in surgical practices [68, 69]. The MicroCutter was used to transect and anastomose a large variety of tissues ranging from the stomach along the entire intestine to the rectum.

**Fig. 6:**
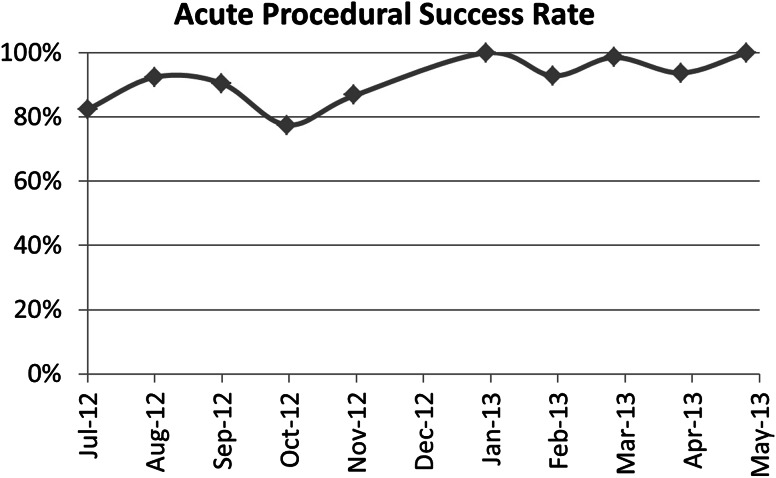
Rate of instrument failures over the enrollment period

As with any mechanical device, there is a learning curve for both device performance and user interaction. The majority of device problems occurred intraoperatively and were readily addressed using conventional surgical techniques without any impairment to the subject or change in the planned procedure. During the study, there were 37 out of 423 deployments (8.7 %) that had a deficient staple line. Each of these “incomplete” deployments was carefully investigated using feedback from the user, video analysis of the deployments (when available), and from subsequent analysis of the device and/or cartridges after they were returned to the manufacturer (available in 36 of 37 deployments (97.2 %). Of the 37 incidences, 22 events (5.2 % of total deployments) were determined to be device related and 15 events (3.5 % of total deployments) considered user error. During this study, several improvements in the device were implemented to improve its functionality and address the issues identified during the study. For example, as the most frequent causes for device failures was related to the use of the stapler in tissue thicker than indicated for a “blue” cartridge, a new version of the MicroCutter including a mechanism to prevent stapler firing if the clamped tissue was too thick was introduced. These changes had a positive impact on the procedure success rate over the course of the enrollment period as shown by a monthly acute procedural success rate, defined as the number of staple firings that met all acute procedural success criteria divided by the total number of deployments attempted that month (Fig. 6).

This documents that product improvements performed “on the fly,” were effective in resolving the issues identified and had a positive effect on the acute procedural success rate.

Although not a formal study endpoint, we were also interested to determine whether there were advantages to this new stapler in general surgical practice. The much smaller shaft diameter (MicroCutter 5 mm versus the 12-mm shaft diameter of conventional staplers) and significantly increased articulation angle (MicroCutter 80° vs 45° of conventional staplers) might be expected to offer several clinical advantages: Based on the experiences gained during the trial, the clinicians involved pointed out several advantages which varied depending on the type of procedure performed.

In appendectomies, the biggest advantage seems to be the fact that the MicroCutter allows the surgeon to perform the procedure with only 5-mm trocars avoiding 12-mm trocars and the associated risk of herniation, infection, pain and discomfort. Obviously, there are other alternatives to staplers for performing appendectomy, but when staplers are indicated—for example in gangrenous cases or in children and young adults—it may provide a true clinical advantage [70]. In gastric bypass procedures, the MicroCutter was predominantly used for the jejunostomy where it was found to significantly reduce the size of the enterotomies needed to insert the stapler jaws. The result is that the common enterotomy is significantly smaller and easier to close, saving time and perhaps reducing the risk of stenosis [9, 71].

The experience in rectal resections was fairly limited. If the thickness of the rectum wall is within the capable range of the MicroCutter, then the ability to articulate to 80 degrees could be a major advantage for the surgeon because it allows a right-angled transection deep in the pelvis [72]. In laparotomy, the smaller shaft diameter allowed the surgeon to get closer to the desired margin without the need to resect excess tissue.

A weakness of the study is that it was not randomized. Rather, comparison to traditional staplers was made by performing a meta-analysis of the complication rates associated with standard staplers and comparing it to the data collected prospectively. The 17 % complication rate we found in the literature may seem high, but as we were only looking for non-inferiority for this new stapler, the absolute number is probably less important than the low incidence of problems documented with the new stapler. We recognize as well that often these staple line failures are sometimes the fault of the stapler, sometimes a factor of patient biology and sometimes multifactorial. As it is impossible in the literature or even clinically to determine whether failure is a mechanism problem or not, we choose to also record all adverse outcomes for the MicroCutter staple lines as well—to ensure fair comparisons. Another weakness is the relatively low percentage of anastomoses done with the new stapler. This was purely the result of the case mix and not by design. Surgeons are understandably concerned in particular about anastomotic integrity, and while this study confirmed the reliability and safety of the MicroCutter in general, it may be worthwhile in the future to do a prospective study just comparing new and traditional staplers in the creation of intestinal anastomoses.

It certainly seems like the new stapler is safe and effective. It should be noted that the study population included uses of both standard staplers and the 5-mm stapler in 42 % of the patients. In this subset of our study population, there was a 10 % incidence of complications with standard staplers versus the <1 % for the MicroCutter. While not truly comparable as the larger staplers were often used for thicker tissues, it may be clinically relevant as the surgeons used the stapler they felt was most relevant for the tissue to be divided. Therefore, this result does tend to validate our historical comparison.

We show safety and efficacy of a novel 5-mm-diameter laparoscopic linear cutting stapler in 160 clinical operations. In over 420 clinical applications of the device, the device was documented to perform well and with few staple line problems (4 %). Thirty-day complications related to the new stapler were very rare (0.65 %) and consisted of a postoperative staple line bleed on POD 1. This compares well with the clinical efficacy data regarding traditional 12-mm staplers which is as high as 17 %.

## Footnotes

1 Excluding subjects without severe adverse events that did not complete 30-day follow-up (*n* = 7).
